# Comparison of crystalloid and colloid co-load combined with norepinephrine prophylaxis on post-spinal anesthesia hypotension during cesarean delivery: a randomized sequential allocation dose-finding study

**DOI:** 10.3389/fmed.2023.1214598

**Published:** 2023-09-01

**Authors:** Yi Chen, Xiangzhao Xu, Rui Qin, Lei Guo, Xinli Ni

**Affiliations:** ^1^Department of Anesthesiology and Perioperative Medicine, General Hospital of Ningxia Medical University, Yinchuan, Ningxia, China; ^2^Department of Anesthesiology, The People’s Hospital of Nanchuan, Chongqing, China

**Keywords:** crystalloid, colloid, co-load, norepinephrine, post-spinal anesthesia hypotension, cesarean delivery

## Abstract

**Background:**

Fluid loading is an essential component of treatment for reducing the incidence of post-spinal anesthesia hypotension and is necessary to maintain intravascular volume, perfuse tissues, and control spinal anesthesia hypotension after sympathetic blockade. We performed a randomized sequential allocation dose-finding study to compare the effects of 10 mL/kg crystalloid and 6% hydroxyethyl starch (130/0.4) co-load on the ED90 of prophylactic norepinephrine infusion for preventing post-spinal anesthesia hypotension during cesarean delivery.

**Methods:**

Eighty patients were randomly allocated to receive either a 10 mL/kg crystalloid (Crystalloid Group, *n* = 40) or 6% hydroxyethyl starch (130/0.4) (Colloid Group, *n* = 40) co-load combined with prophylactic norepinephrine infusion during spinal anesthesia for cesarean delivery. The first patient received an initial prophylactic norepinephrine infusion rate of 0.025 μg/kg/min. Subsequent patients received a 0.005 μg/kg/min gradient dose of prophylactic norepinephrine. This dose was administered as a gradient based on its effectiveness for preventing post-spinal anesthesia hypotension (defined as SBP < 80% of baseline value) and determined by the up-and-down sequential allocation methodology. The primary study outcome was the ED90 of prophylactic norepinephrine infusion. Secondary outcomes included the incidence of post-spinal anesthesia hypotension, bradycardia, hypertension, Apgar scores, and umbilical artery blood gas values were also measured.

**Results:**

The ED90 values of prophylactic norepinephrine infusion for preventing post-spinal anesthesia hypotension during cesarean delivery were 0.063 μg (95% CI: 0.050 to 0.064) and 0.062 μg (95% CI: 0.045 to 0.064) using isotonic regression analysis, and 0.068 μg (95% CI: 0.056 to 0.353) and 0.060 μg (95% CI: 0.050 to 3.590) using probit regression analysis in the Crystalloid Group and Colloid Group, respectively. The secondary outcomes were comparable between the two groups.

**Conclusion:**

The administration of a 10 mL/kg 6% hydroxyethyl starch (130/0.4) does not provide additional benefits compared to crystalloid co-load in reducing the ED90 of prophylactic norepinephrine infusion for preventing post-spinal anesthesia hypotension during cesarean delivery.

## 1. Introduction

Spinal anesthesia is the preferred method of anesthesia for cesarean delivery, but it may contribute to post-spinal anesthesia hypotension, the most common maternal adverse event. Post-spinal anesthesia hypotension can result in complications, including nausea, vomiting, decreased blood flow to the placenta, and fetal acidosis (1). Vasopressors and fluid loading are vital for preventing and treating post-spinal anesthesia hypotension, as they improve hemodynamic stability (2, 3). Fluid loading is an essential component of treatment for reducing the incidence of post-spinal anesthesia hypotension and is necessary to maintain intravascular volume, perfuse tissues, and control spinal anesthesia hypotension after sympathetic blockade (4). Further, fluid loading reduces the requirement for vasopressors and improves the hemodynamic stability conferred by prophylactic vasopressor infusion (2, 3, 5).

Pre-load and co-load are the two most commonly used methods of fluid loading to reduce the incidence of post-spinal anesthesia hypotension and improve hemodynamic stability (6, 7). Previous literature suggests co-load is more effective than pre-load and colloid is superior to crystalloid (5, 8). A recently adopted strategy for mitigating post-spinal anesthesia hypotension is the administration of a 10 mL/kg co-load of either colloid or crystalloid in combination with a prophylactic vasopressor infusion (6, 9–11). However, the relative benefits of using colloid versus crystalloid in combination with a prophylactic norepinephrine infusion at this dosage, and the influence of different types of fluid on the effective dose (ED) 90 of prophylactic norepinephrine infusion, remain unclear. Therefore, we performed a randomized sequential allocation dose-finding study to determine whether a 10 mL/kg crystalloid or 6% hydroxyethyl starch (130/0.4) co-load affected the ED90 of prophylactic norepinephrine infusion required for preventing post-spinal anesthesia hypotension during cesarean delivery.

## 2. Materials and methods

This study was conducted in accordance with the Declaration of Helsinki and approved by the Ethics Committee at the General Hospital of Ningxia Medical University, Yinchuan City, Ningxia Province, China (No. KYLL-2023-0053) and the Ethics Committee of The People’s Hospital of Nanchuan, Chongqing City, China (No. QT-2023-003) from January 2023 to March 2023. The study was registered at www.clinicaltrials.gov (NCT05475990) and all patients provided written informed consent before recruitment.

### 2.1. Study population

We recruited women aged 18–40 years with single term pregnancy who were scheduled for elective cesarean delivery under spinal anesthesia. Patients with a body mass index ≥40 kg/m^2^, preeclampsia or pre-existing pregnancy-induced hypertension, known allergy or contraindication to hydroxyethyl starch, fetal distress, or American Society of Anesthesiologists physical status ≥III were excluded.

### 2.2. Monitoring and anesthesia

Baseline systolic blood pressure (SBP) and heart rate (HR) were recorded for three consecutive readings at resting state. SBP and HR were then recorded every minute for 15 min after induction of spinal anesthesia, and then every 5 min until the end of the operation. Norepinephrine, crystalloid, and 6% hydroxyethyl starch (130/0.4) were infused through an 18-G intravenous (IV) catheter without any preload before spinal anesthesia. A 12.5-mg hyperbaric bupivacaine solution (0.5% w/v) was injected using a 25-G spinal needle at the L3-4 vertebral interspace for spinal anesthesia. Patients were positioned supine with left lateral tilt, and the operation was permitted to proceed if the sensory block (assessed by a sterile needle) was greater than T6.

### 2.3. Study protocol

Immediately after spinal anesthesia, a 10 mL/kg crystalloid [compound sodium chloride (0.85% NaCl, 0.03% KCl, and 0.033% CaCl_2_)] or 6% hydroxyethyl starch (130/0.4) co-load was infused over 10–15 min with a pressure bag according to group allocation (Crystalloid Group or Colloid Group; as assigned by a computer-generated sequence for randomization placed in sealed, opaque envelopes). Crystalloid was then maintained at a rate of 6 mL/kg/h to a maximum liquid volume of 2 L until the end of the operation. Patients and researchers, but not the anesthesiologist, were blinded to the type of fluid used.

The first patient received an initial dose of prophylactic norepinephrine infusion at a rate of 0.025 μg/kg/min simultaneously with spinal anesthesia in both the Crystalloid and Colloid Groups. A 0.005 μg/kg/min dose of prophylactic norepinephrine was administered as a gradient based on its effectiveness for preventing post-spinal anesthesia hypotension (defined as SBP <80% of baseline value) and determined by the up-and-down sequential allocation methodology. If hypotension was successfully prevented for three consecutive patients, the gradient of 0.005 μg/kg/min prophylactic norepinephrine was decreased. If it failed to prevent hypotension, the gradient of 0.005 μg/kg/min prophylactic norepinephrine was increased for the next patient.

### 2.4. Outcome measurement

The primary outcome was the dose of prophylactic norepinephrine infusion required to prevent post-spinal anesthesia hypotension in 90% (ED 90) of patients. Post-spinal anesthesia hypotension was defined as an SBP reading <80% of baseline within 15 min following spinal anesthesia, and severe post-spinal anesthesia hypotension was defined as an SBP reading <60% of baseline within the same time frame. If post-spinal anesthesia hypotension was detected, a 6-μg norepinephrine IV bolus was given. If an SBP reading was >120% of baseline (defined as hypertension), norepinephrine infusion was discontinued until SBP returned to <120% of baseline. Bradycardia was defined as a HR of <60 beats/min, and treated with 0.5 mg IV atropine. Apgar scores at 1 min and 5 min after birth and umbilical artery blood gas values were also measured.

### 2.5. Sample size determination and statistical analysis

To achieve a stable estimation of ED using the up-and-down method, a sample size of 20–40 subjects was recommended (12). We allocated 40 patients each to a Crystalloid and a Colloid Group.

The normality of distribution for continuous variables was assessed using the Shapiro–Wilk test. Normally distributed continuous variables were presented as mean ± SD and analyzed using an unpaired t-test. Non-normally distributed continuous variables were presented as median (interquartile range, IQR) and analyzed using a Mann–Whitney U test. Categorical variables were presented as number (%) and analyzed using a Chi-square test. The ED90 of prophylactic norepinephrine infusion for preventing post-spinal anesthesia hypotension were determined using isotonic regression analysis and probit regression analysis. IBM SPSS Statistics 22.0 (IBM Corp, Armonk, NY) was used for data analysis, and *p* < 0.05 was considered significant.

## 3. Results

**Figure 1:**
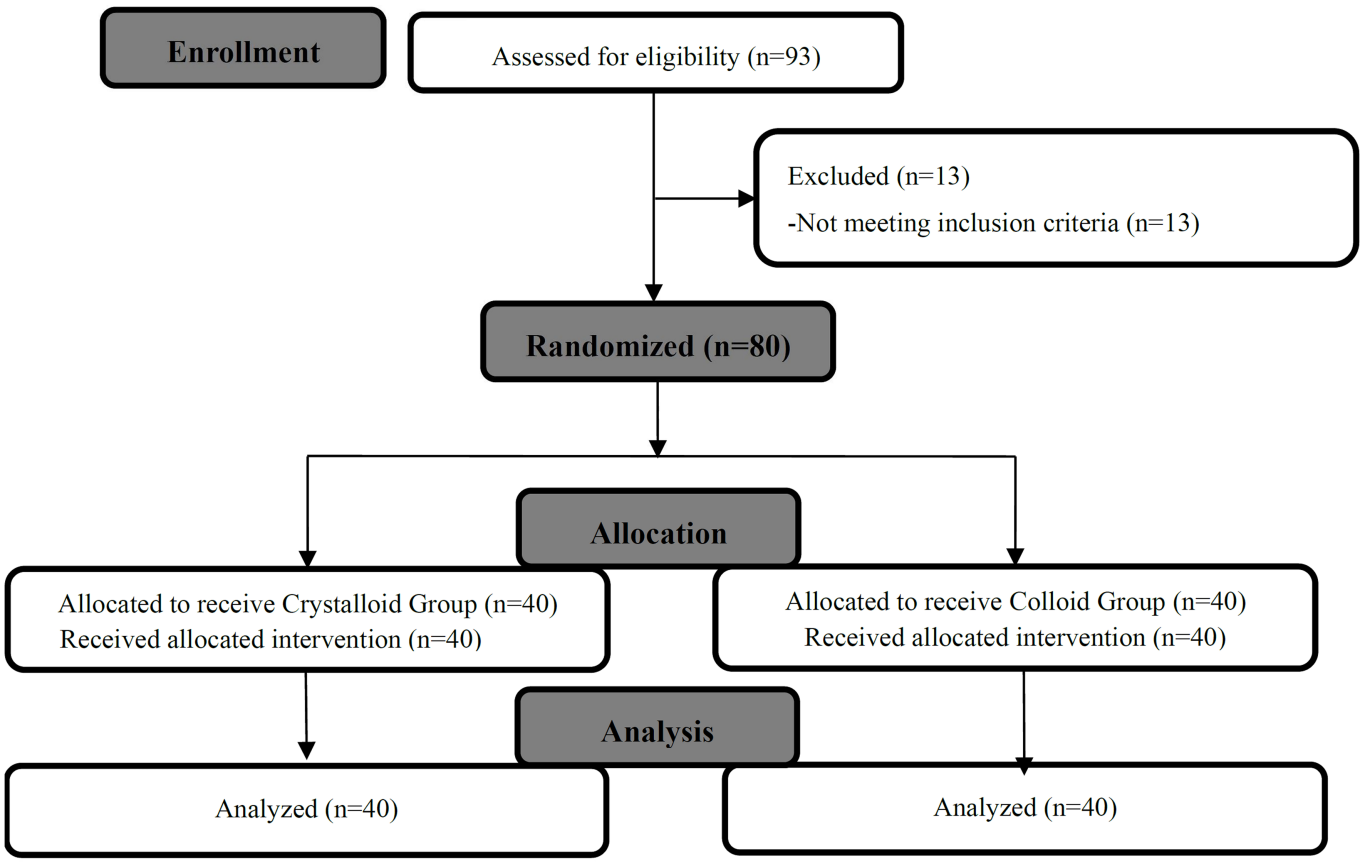
Study flow diagram.

We randomly allocated and analyzed 40 patients in the Crystalloid Group and 40 patients in the Colloid Group. A patient flow chart is shown in Figure 1. Patient demographics, baseline characteristics, and surgical times were comparable between the two groups, as shown in Table 1.

**Table 1:** Patient characteristics.

	Crystalloid Group (*n* = 40)	Colloid Group (*n* = 40)	*p* value
Age (yr)	32.35 ± 4.79	31.93 ± 4.70	0.690
BMI (kg/m^2^)	27.89 ± 2.96	28.42 ± 3.77	0.492
Baseline characteristics			
Systolic blood pressure (mmHg)	119.10 ± 12.14	116.00 ± 7.64	0.176
Heart rate (beats/min)	89.98 ± 9.06	93.90 ± 12.02	0.103
Block height	T6 (T4 - T6)	T6 (T5 - T6)	0.103
Spinal anesthesia to fetal delivery interval (min)	14.55 ± 2.28	15.53 ± 2.67	0.083
Skin incision to fetal delivery interval (min)	2.75 ± 1.15	3.15 ± 1.53	0.190
Length of postoperative stay (d)	3.30 ± 0.52	3.45 ± 0.64	0.252

Data are showed as mean ± SD and median (IQR). BMI, body mass index.

### 3.1. The sequences of patients with effective or ineffective responses and the primary outcome

**Figure 2:**
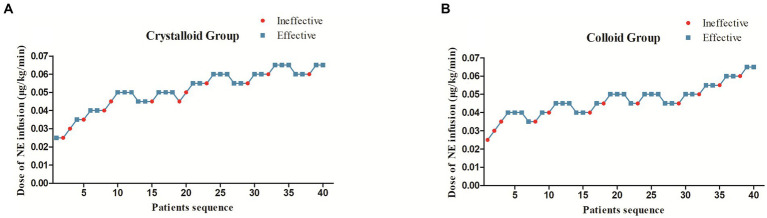
The sequence of patients with effective or ineffective responses to the prophylactic norepinephrine infusion in the Crystalloid Group **(A)** and Colloid Group **(B)**.

**Figure 3:**
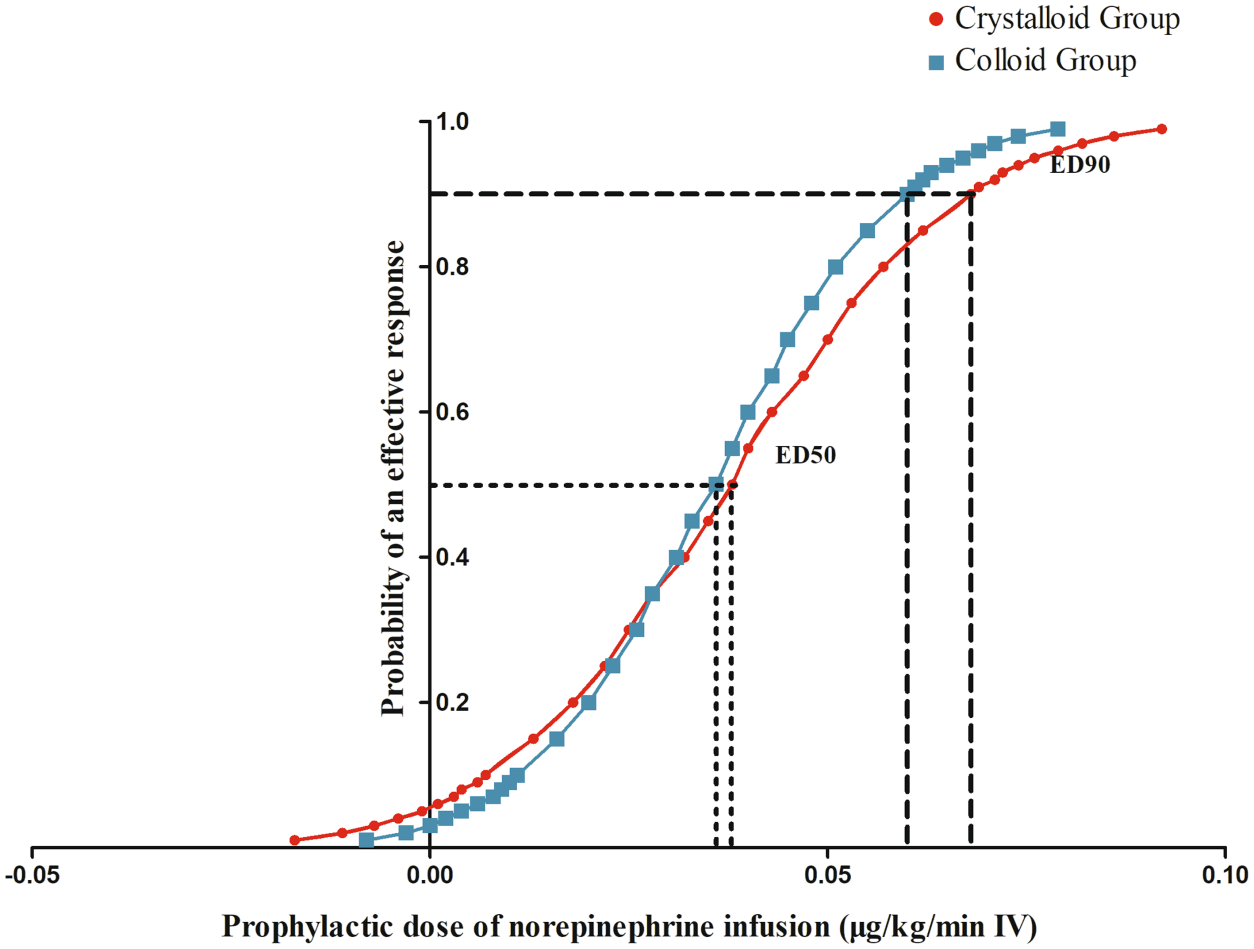
The dose–response curves of prophylactic norepinephrine infusion for preventing post-spinal anesthesia hypotension.

The sequences of patients with effective or ineffective responses to the prophylactic norepinephrine infusion in each group are shown in Figures 2A,B. The dose–response curves of prophylactic norepinephrine infusion using probit regression analysis are shown in Figure 3. The estimated values for the ED90 of prophylactic norepinephrine infusion for preventing post-spinal anesthesia hypotension were determined to be 0.063 μg (95% CI: 0.050 to 0.064) and 0.062 μg (95% CI: 0.045 to 0.064) using isotonic regression analysis, and 0.068 μg (95% CI: 0.056 to 0.353) and 0.060 μg (95% CI: 0.050 to 3.590) using probit regression analysis in the Crystalloid Group and Colloid Group, respectively. The response rates for prophylactic norepinephrine infusion doses are shown in Table 2.

**Table 2:** Response rates for doses of prophylactic norepinephrine infusion.

Assigned dose of norepinephrine (ug)	Crystalloid Group (*n* = 40)			Colloid Group (*n* = 40)		
	Number of successes	Number of patients	Response rate (%)	Number of successes	Number of patients	Response rate (%)
0.025	1	2	50.00%	0	1	0.00%
0.03	0	1	0.00%	0	1	0.00%
0.035	1	2	50.00%	1	3	33.33%
0.04	2	3	66.67%	6	8	75.00%
0.045	2	5	40.00%	7	10	70.00%
0.05	6	7	85.71%	8	9	88.89%
0.055	4	6	66.67%	2	3	66.67%
0.06	7	9	77.78%	2	3	66.67%
0.065	5	5	100.00%	2	2	100.00%

### 3.2. Maternal and neonatal outcomes

Maternal and neonatal outcomes were not different between the two groups, as shown in Table 3.

**Table 3:** Maternal and neonatal outcomes.

	Crystalloid Group (*n* = 40)	Colloid Group (*n* = 40)	*p* value
Postspinal anesthesia hypotension, *n* (%)	12 (30.0)	14 (35.0)	0.406
Severe postspinal anesthesia hypotension, *n* (%)	1 (2.5)	1 (2.5)	1.000
Bradycardia, *n* (%)	1 (2.5)	3 (7.5)	0.615
Nausea, *n* (%)	6 (15.0)	5 (12.5)	0.500
Vomiting, *n* (%)	4 (10.0)	3 (7.5)	0.500
Hypertension, *n* (%)	1 (2.5)	2 (5.0)	0.500
pH	7.35 ± 0.04	7.35 ± 0.03	0.927
pH < 7.2, *n* (%)	0 (0.0)	0 (0.0)	1.000
PCO_2_ (mmHg)	42.74 ± 4.73	41.56 ± 4.08	0.242
PO_2_ (mmHg)	21.97 ± 5.21	22.78 ± 4.93	0.484
BE (mmol/L)	−2.06 ± 1.63	−2.28 ± 1.35	0.531
Apgar score, 1 min	9 (9–9)	9(9–9)	0.366
<7 at 1 min, *n* (%)	0 (0)	0 (0)	1.000
Apgar score, 5 min	10 (10–10)	10 (10–10)	0.242
<7 at 5 min, *n* (%)	0 (0)	0 (0)	1.000

Data are showed as number (%), mean ± SD and median (IQR). PCO2, partial pressure of carbon dioxide; BE, base excess; PO2, partial pressure of oxygen.

## 4. Discussion

In this randomized sequential allocation dose-finding study, we compared the effect of crystalloid co-load and 6% hydroxyethyl starch (130/0.4) co-load on the ED90 of prophylactic norepinephrine infusion for preventing post-spinal anesthesia hypotension during cesarean delivery. We found that the administration of either 10 mL/kg of 6% hydroxyethyl starch (130/0.4) or crystalloid co-load demonstrated comparable efficacy in reducing ED90 of prophylactic norepinephrine infusion for preventing post-spinal anesthesia hypotension during cesarean delivery.

Previous studies have also investigated the effect of different fluid loading protocols on the incidence and severity of post-spinal anesthesia hypotension. Jin et al. compared the effect of 10 mL/kg crystalloid and 6% hydroxyethyl starch (130/0.4) co-load on the ED50 and ED90 of different doses of prophylactic norepinephrine infusion (0.02, 0.04, 0.06, 0.08 and 0.01 ug/kg/min), and found that the ED90 of crystalloid and colloid co-load were 0.097 μg (95% CI: 0.072 to 0.157 μg) and 0.070 μg (95% CI: 0.053 to 0.107 μg), respectively. They determined that colloid co-load reduced the dose of prophylactic norepinephrine infusion required to prevent post-spinal anesthesia hypotension by approximately 30% (6). Xu et al. evaluated the ED95 of a range of prophylactic norepinephrine infusion doses (0, 0.025, 0.05, 0.075, and 0.1 ug/kg/min) combined with 10 mL/kg crystalloid co-load and found that the ED95 was 0.097 μg/kg/min (95% CI: 0.081 to 0.134 μg/kg/min) (13). In our study, the observed ED90 in both the Crystalloid and Colloid Groups was lower than that reported by Jin et al. (6). The different doses of prophylactic norepinephrine infusion used in the randomized controlled trial reported by both studies (6, 13) may have contributed to the observed difference in ED90 relative to our study, which used an up-and-down sequential allocation methodology.

Iatrogenic sympathetic block following spinal anesthesia contributes to rapid arterial vasodilation, leading to a decrease in peripheral vascular resistance, reducing preload and afterload, and causing a compensatory increase in cardiac output (14). The severity of these cardiovascular effects is influenced by block height, the patient’s position, and preventive measures (15). Uteroplacental perfusion, which lacks autoregulation, is highly dependent on maternal cardiac output and, under spinal anesthesia, a decrease in cardiac output correlates with fetal acidosis (16). Norepinephrine, a drug with mixed potent α and weak β activity, is commonly used for obstetric anesthesia as an alternative to phenylephrine. Notably, norepinephrine may improve maternal hemodynamic stability and cardiac output while avoiding bradycardia (12).

Fluid loading, either alone or in combination with vasopressors, has been identified as an important strategy for preventing and treating post-spinal anesthesia hypotension (17). Different protocols of fluid loading, including types (crystalloid or colloid), timing (preload or co-load), and volume, have been shown to reduce, but not prevent, post-spinal anesthesia hypotension, therefore, the combined administration of vasopressors may be more crucial (18). However, our findings suggest that colloid co-load may not offer additional benefits when combined with prophylactic norepinephrine infusion, compared to crystalloid. Consequently, crystalloid may be the preferred option for administering relatively large co-load doses of 10 mL/kg or higher. Although crystalloid preload alone may offset hypovolemia related to pre-operative fasting and significantly increase cardiac output before spinal anesthesia, it is insufficient for maintaining SBP and corrected flow time or reducing post-spinal anesthesia hypotension incidence (19, 20). Conversely, crystalloid co-load provides better intravascular volume expansion with less rapid redistribution (21). However, due to their larger molecular weight, colloids are unable to easily cross into the interstitial fluid and remain in the intervascular space for a longer duration relative to crystalloids, promoting superior intravascular volume expansion and increased osmotic pressure (22, 23). In a recent study, Theodoraki et al. demonstrated that the incidence of post-spinal anesthesia hypotension was low and comparable between colloid preload and crystalloid co-load when combined with prophylactic norepinephrine infusion (24). This result indicates the potential advantages of administering crystalloid.

Before the hemodynamic changes induced by sympathetic blockade, excessive fluid would rapidly redistribute into the interstitial fluid (25). The administration of a rapid crystalloid preload may contribute to the distension of atrial chamber and subsequent stimulation of atrial natriuretic peptide secretion, resulting in peripheral vasodilation and a diuretic effect (25). Conversely, co-load may result in a lesser aforementioned effect and restrict the redistribution of fluid loading into the interstitial compartment due to the decrease in hydrostatic pressure, concomitant with spinal-induced vasodilation (6, 24). Though co-load can help reduce the incidence and severity of post-spinal anesthesia hypotension, additional vasopressors are commonly required to counter arteriolar vasodilation (6). Thus, vasopressors are consistently considered the preferred strategy for preventing and treating post-spinal anesthesia hypotension. Further, co-loading does not require delaying the induction of spinal anesthesia or the operation, especially for an emergency delivery. However, the effectiveness of a co-load may be limited by its slower infusion speed (15), necessitating the use of a high-flow IV catheter and pressure bag.

Our study has some important limitations. Though the risk of allergic reactions is reduced with the use of 6% hydroxyethyl starch (130/0.4) compared to dextran and gelatin, its use is associated with the risk of coagulopathy and renal damage. Larger co-load volumes, such as 15 mL/kg (26) were not evaluated in our study and may also influence the ED90 of prophylactic norepinephrine infusion for preventing post-spinal anesthesia hypotension. The use of larger volumes may not result in additional benefits and may increase the risk of adverse events (27). The optimal volume of crystalloid or colloid co-load should be explored in future studies. Notably, few studies have investigated the effect of larger colloid volumes on puerperal outcomes for cesarean delivery, including maternal and neonatal adverse events and placental transfer. However, a Cochrane review indicated that there is little difference in mortality within 30 and 90 days between the administration of crystalloid and colloid (23). We did not perform direct measurements of cardiac output, such as cardiac output and stroke volume variation, which could provide more precise guidance for maternal hemodynamics and fluid management. Additionally, we excluded patients with comorbidities and fetal compromise from our study, and the attending anesthesiologist was not blinded to the type of fluid administered, which could potentially influence study outcomes.

In conclusion, the combined strategy of administering fluid loading and vasopressor represents an effective approach for preventing post-spinal anesthesia hypotension. The administration of a 10 mL/kg 6% hydroxyethyl starch (130/0.4) does not provide additional benefits compared to crystalloid co-load in reducing the ED90 of prophylactic norepinephrine infusion for preventing post-spinal anesthesia hypotension during cesarean delivery.

## Data availability statement

The original contributions presented in the study are included in the article/supplementary materials, further inquiries can be directed to the corresponding author.

## Ethics statement

The studies involving humans were approved by The Ethics Committee at the General Hospital of Ningxia Medical University, Yinchuan City, Ningxia Province, China (No. KYLL-2023-0053) and the Ethics Committee of The People’s Hospital of Nanchuan, Chongqing City, China (No. QT-2023-003). The studies were conducted in accordance with the local legislation and institutional requirements. The participants provided their written informed consent to participate in this study.

## Author contributions

YC, XX, RQ, LG, and XN helped in designing and conducting the study, analyzing the data, and preparing the manuscript. All authors contributed to the article and approved the submitted version.

## Funding

This work was supported by the Ningxia Natural Science Foundation (No. 2022AAC03591) and the Ningxia Health System Fund for Scientific Research (No. 2022-NWKY-056).

## Conflict of interest

The authors declare that the research was conducted in the absence of any commercial or financial relationships that could be construed as a potential conflict of interest.

## Publisher’s note

All claims expressed in this article are solely those of the authors and do not necessarily represent those of their affiliated organizations, or those of the publisher, the editors and the reviewers. Any product that may be evaluated in this article, or claim that may be made by its manufacturer, is not guaranteed or endorsed by the publisher.
